# Investigating cognitive flexibility deficit in schizophrenia using task-based whole-brain functional connectivity

**DOI:** 10.3389/fpsyt.2022.1069036

**Published:** 2022-11-21

**Authors:** Yanqing Wang, Xueping Hu, Yilu Li

**Affiliations:** ^1^Institute of Psychology, Chinese Academy of Sciences, Beijing, China; ^2^Department of Psychology, University of Chinese Academy of Sciences, Beijing, China; ^3^School of Linguistic Science and Art, Jiangsu Normal University, Xuzhou, China; ^4^Key Laboratory of Language and Cognitive Neuroscience of Jiangsu Province, Collaborative Innovation Center for Language Ability, Xuzhou, China; ^5^The Clinical Hospital of Chengdu Brain Science Institute, MOE Key Laboratory for Neuroinformation, Center for Information in Medicine, School of Life Science and Technology, University of Electronic Science and Technology of China, Chengdu, China

**Keywords:** schizophrenia, cognitive flexibility, functional connectivity, multivariate pattern analysis, graph theory analysis

## Abstract

**Background:**

Cognitive flexibility is a core cognitive control function supported by the brain networks of the whole-brain. Schizophrenic patients show deficits in cognitive flexibility in conditions such as task-switching. A large number of neuroimaging studies have revealed abnormalities in local brain activations associated with deficits in cognitive flexibility in schizophrenia, but the relationship between impaired cognitive flexibility and the whole-brain functional connectivity (FC) pattern is unclear.

**Method:**

We investigated the task-based functional connectivity of the whole-brain in patients with schizophrenia and healthy controls during task-switching. Multivariate pattern analysis (MVPA) was utilized to investigate whether the FC pattern can be used as a feature to discriminate schizophrenia patients from healthy controls. Graph theory analysis was further used to quantify the degrees of integration and segregation in the whole-brain networks to interpret the different reconfiguration patterns of brain networks in schizophrenia patients and healthy controls.

**Results:**

The results showed that the FC pattern classified schizophrenia patients and healthy controls with significant accuracy. Moreover, the altered whole-brain functional connectivity pattern was driven by a lower degree of network integration and segregation in schizophrenia, indicating that both global and local information transfers at the entire-network level were less efficient in schizophrenia patients than in healthy controls during task-switching processing.

**Conclusion:**

These results investigated the group differences in FC profiles during task-switching and not only elucidated that FC patterns are changed in schizophrenic patients, suggesting that task-based FC could be used as a potential neuromarker to discriminate schizophrenia patients from healthy controls in cognitive flexibility but also provide increased insight into the brain network organization that may contribute to impaired cognitive flexibility.

## Introduction

Cognitive flexibility, the ability to adjust one’s behavior or cognitive action in response to changing environmental demands, is considered a core cognitive control function (1, 2). The performance of task-switching is an important measure of cognitive flexibility. In task-switching, participants randomly alternate between the performance of two (or more) tasks, with an advance cue specifying the task to perform on the upcoming trial.

Schizophrenia is a mental disorder with deficits in cognitive flexibility (1, 3). Specifically, people with schizophrenia need a longer time to disengage from the previous task set and engage in an upcoming task set, as evidenced by longer response times and higher error rates compared to healthy controls in task-switching tasks (4–6). In parallel, reduced activation in the lateral prefrontal cortex anterior cingulate cortex and in schizophrenia has been found among several cognitive flexibility-related tasks (7–10).

Previous studies have mainly concentrated on the variation in local brain activations associated with deficits in cognitive flexibility in schizophrenia. Notably, recent research has suggested that the neural activities recorded during the performance of a task are reflected not only by a change in neural activity in specific regions of the brain but also by an interaction across large-scale brain systems *via* region activity (11–14). Moreover, given that task-switching consists of a series of functions, including selecting goal-relevant information, maintaining goal-relevant information, and inhibiting goal-irrelevant information (15–17), logically, it involves a wide variety of brain regions. Accordingly, whole-brain measures should provide a more comprehensive measure of cognitive flexibility than activity in a single brain region. In addition, a prominent hypothesis for the origin of cognitive deficits in schizophrenia is that of anatomical and functional brain dysconnectivity at multiple scales of space and time, ranging from neurochemical dysconnectivity to emerged functional dysconnectivity (18, 19). This hypothesis posits that schizophrenia can be conceived as a disorder of connectivity between large-scale brain networks (19–21). Using resting state functional magnetic resonance imaging (fMRI), increasing evidence has shown that alterations in whole-brain functional connectivity (FC) are reliable predictors of cognitive changes related to brain diseases including schizophrenia (12, 13, 22, 23). Most previous studies using FC analysis methods have been conducted with participants during the resting state, while how the task-modulated connectivity of large-scale brain networks is altered in schizophrenia during task-switching tasks and whether the altered FC could be used to distinguish schizophrenic patients from healthy controls remains largely unknown.

Motivated by the aforementioned consideration, we analyzed the fMRI data of the task-switching task to determine how FC is altered in schizophrenia patients relative to healthy controls during the switch condition. Specifically, the beta series correlation technique (24) was first performed to analyze the FC between schizophrenia patients and healthy controls during task-switching. An increasing number of studies have shown that the brain functions of network differences between patients with psychiatric disorders and healthy controls were determined by its connectivity patterns rather than connection strength (13, 22). Multivariate pattern analysis (MVPA), a method sensitive to fine-grained spatial discriminative patterns and the exploration of the inherent multivariate nature from high-dimensional neuroimaging data, was then conducted to classify the schizophrenia and control groups and identify FC differences.

Moreover, recent studies using graph-theoretical analysis methods suggested that patients with schizophrenia during resting state present abnormalities in topological properties of the brain network connectivity, including less hierarchical, less small-world, less clustered, and less efficient connectivity (19, 25–27). These differences might be expected to impair higher-order cognitive functions (28). For example, the task-induced reconfiguration of FC during an auditory oddball task is characterized by less immediate communication between nodes in schizophrenia vs. controls (29). Accordingly, we infer that the abnormal FC patterns during task-switching in schizophrenic patients are caused by abnormal topological properties. To assess the different topological properties of brain networks in schizophrenia patients and healthy controls in cognitive flexibility, we then calculated the degrees of integration and segregation in the whole-brain networks by using graph theory analysis, which could capture the features of the brain network architecture and was applicable to explore brain network organization in various cognitive processes (30, 31).

## Materials and methods

### Participants

Data used in this study were obtained from the OpenNeuro database with accession number of ds000030. The dataset contains multimodal brain imaging and behavioral data from patients with schizophrenia (*n* = 50) and healthy controls (*n* = 130). Diagnoses were based on the Diagnostic and Statistical Manual of Mental Disorders, Fourth Edition-Text Revision (DSM-IV) and per the Structured Clinical Interview for DSM-IV (SCID-I). All participants gave written informed consent during the data collection of the UCLA Consortium for Neuropsychiatric Phenomics LA5c Study. More information about participant and study procedures can be found in the corresponding data paper (32). After removing participants with missing files and large head motions (total displacement > 3 mm), fMRI data from a sex- and age-matched subset of the healthy control (*n* = 46, mean age = 36.35 years, SD = 8.71, 14 females) and schizophrenia (*n* = 46, mean age = 36.54 years, SD = 8.95, 14 females) cohorts were used in the final analysis.

### Task-switching task

During cued task-switching, participants were cued to perform one of two alternative tasks (shape task vs. color task) on each trial. In the shape task, the cues presented included either “SHAPE” or “S” on trials where participants had to decide if the shape feature of the stimulus was a circle or triangle. In the color task, the cues presented included either “COLOR” or “C” on trials where participants had to decide whether the color feature of the stimulus was red or green. In total, 96 trials presented in a pseudorandomized order, including 24 switch trials (where the cued task for the current trial differed from that on the previous trial) and 72 repeat trials (where the cued task for the current trial was the same as that on the previous trial).

### Data acquisition and preprocessing

Functional magnetic resonance imaging data were collected using a Siemens Trio 3 T scanner and a Siemens 32-channel head coil. Functional images were acquired using echo-planar imaging with the following parameters: *TR* = 2,000 ms, *TE* = 30 ms, flip angle = 90°, acquisition matrix = 64 × 64, slice number = 34. T1-weighted scans were acquired with the following parameters: *TR* = 1,900 ms, TE = 2.26 ms, acquisition matrix = 256 × 256, slice number = 176.

The preprocessing of fMRI data was done using the SPM12 software^1^ on the MATLAB platform. Functional images were preprocessed for slice-timing correction, motion correction (realignment), coregistration, gray/white matter segmentation, normalization to the MNI template and spatial smoothing using a 6 mm full-width half-maximum Gaussian kernel.

### Brain network partition

The whole-brain was divided into 264 regions of interest (ROIs) according to the Power atlas (33). This network partition has been shown to provide higher test–retest reliability for brain network properties. Cole et al. assigned 227 of the original 264 ROIs from Power et al. to 10 different brain networks (34). On the basis of Cole et al. five nodes were eliminated due to low signal, and the other 222 ROIs were used in the subsequent analyses. For each of these 222 ROIs, a sphere with a radius of 6 mm was defined.

### Functional connectivity

For connectivity analysis, we obtained the beta map for each trial and estimated the correlations of trial-by-trial variabilities among the 222 ROIs for the switch condition. More specifically, the single-trial response estimations were first performed using the least-square separate method to obtain the beta map for each trial (35). The general linear model (GLM) of a trial included two regressors: The trial of interest and all other trials. Again, stimuli were modeled as stick functions at the onset of cue presentation, and each regressor was convolved with a canonical hemodynamic function. The six motion parameters and the mean time series in the white matter and cerebral spinal fluid were included in each GLM as confounding regressors. After estimating beta maps for different trials, the mean beta values of each ROI were extracted to create a beta series for each participant. Pearson’s correlation coefficients were computed between all pairs of ROIs, resulting in (222 × 221)/2 = 24,531-dimensional FC feature vectors for each participant. These FC feature vectors were used in subsequent analyses.

### Multivariate pattern analysis

A support vector machine (SVM) classifier with a linear kernel and *C* = 1 (36) was applied for classification. The FC vector for each individual was fed into the classification analyses as features. Because the feature size was significantly larger than the sample size, feature reduction was first carried out to prevent overfitting. In this study, we selected features using the *F* score method (37), which has been applied in previous studies and is simple and generally quite effective (13, 38, 39). Specifically, this method selects features that have high similarity within groups and large variance between-groups, as shown in the following.


$$\begin{array}{l} F(i)=\\ \frac{{\left({\bar{x_{i}}}^{\left(+\right)}-\bar{x_{i}}\right)}^{2}+{\left({\bar{x_{i}}}^{\left(-\right)}-\bar{x_{i}}\right)}^{2}}{\frac{1}{n_{+}-1}\sum_{k=1}^{n_{+}}{\left(x_{k,i}^{\left(+\right)}-{\bar{x_{i}}}^{\left(+\right)}\right)}^{2}+\frac{1}{n_{-}-1}\sum_{k=1}^{n_{-}}{\left(x_{k,i}^{\left(-\right)}-{\bar{x_{i}}}^{\left(-\right)}\right)}^{2}} \end{array}$$

where ***x_k_*** represents the training vector containing both positive and negative instances, and n_+_/n_–_ is the number of positive/negative instances. The mean value of the *i*th feature of the whole, positive, and negative datasets are ${\bar{\text{x}}}_{i}$, ${\bar{\text{x}}}_{i}^{\left(+\right)}$, and ${\bar{\text{x}}}_{i}^{\left(-\right)}$; $x_{k,i}^{\left(+\right)}$/$x_{k,i}^{\left(-\right)}$ represents the *i*th feature of the *k*th positive/negative instance. The numerator measures the between-group differences, and the denominator represents the within-group differences. A higher F score indicates that this feature is more discriminative between-groups; hence, this criterion was used for feature selection.

We then applied the leave-one-out cross-validation (LOOCV) method to assess the performance of the classifier (38). For each LOOCV iteration, the *F* score of all 24,531 features was computed and ranked within the training set, where a higher *F* score indicates larger group differences. The feature number was first tested from 20 to 24,520 with a step length of 20. The smallest step that achieved the highest accuracy was chosen, and the corresponding classification results were reported. To test the performance of the classifier, the accuracy, sensitivity, specificity, and area under the curve (AUC) were computed.


$$\text{Accuracy}=\frac{\text{TP+TN}}{\text{TP+FN+TN+FP}}$$

$$\text{Sensitivity}=\frac{\text{TP}}{\text{TP+FN}}$$

$$\text{Specificity}=\frac{\text{TN}}{\text{TN+FP}}$$

Where TP represents the number of schizophrenia patients who were correctly classified; TN represents the number of healthy controls who were correctly classified; FN represents the number of schizophrenia patients who were incorrectly identified as healthy controls; and FP represents the number of healthy controls who were incorrectly identified as schizophrenia patients.

Moreover, the permutation test was employed to measure whether the calculated classification accuracy was statistically significant (40). For each permutation test, labels for the schizophrenia and healthy control groups were shuffled and then replicated the same classification procedure. The permutation test with LOOCV was performed 1,000 times, and the significance was estimated by dividing the number of permutations that displayed a larger value than the actual accuracy by the total number of permutations.

### Graph theory analysis

We performed graph theory analysis to examine the integration and segregation of the brain network by using the GRETNA toolbox.^2^ Using the connectivity matrix obtained in the FC analysis, a weighted, undirected graph was constructed. The weighted network was thresholded at various levels of sparsity (5–50% in 5% increments) to avoid any thresholding bias. The global efficiency (*E*_*g*_) and local efficiency (*E*_*loc*_) were calculated to measure the brain network topologies. In the present study, *N* represents all nodes in the network, and n represents the number of nodes (*i, j*), represents a link between nodes i and j, and (*i, j*?N) and links (*i, j*) are related to the connection weights *w*_*ij*_.

Global efficiency (*E*_*g*_) measures the degree of integration of brain networks. It is the average reciprocal of the shortest path length of all node pairs in the brain network. A larger global efficiency value of the brain network represents a higher information transmission efficiency and a higher the integration degree of the brain network. Global efficiency is defined as follows:


$$E_{g}=\frac{1}{n}\sum_{i\in N}E_{g,i}=\frac{1}{n}\sum_{i\in N}\frac{\sum_{j\in N,j\neq i}{\left(d_{ij}^{w}\right)}^{-1}}{n-1}$$

Where *E*_*g,i*_ represents the efficiency of node *i* and $d_{ij}^{w}$ represents the weighted shortest path length between *i* and *j*.

Local efficiency (*E*_*loc*_) measures the degree of segregation of brain networks. The local efficiency of a node refers to the average reciprocal of the shortest path length of all node pairs in a subgraph composed of the node’s neighbors. The local efficiency of the brain network is the average of the local efficiency of all nodes in the brain network.


$$\begin{array}{l} E_{loc}=\frac{1}{n}\sum_{i\in N}E_{loc,i}\\ =\frac{1}{n}\sum_{i\in N}\frac{\sum_{j,h\in N,j\neq h}{\left(w_{ij}w_{ih}{\left[d_{jh}^{w}(N_{i})\right]}^{-1}\right)}^{1/3}}{k_{i}\left(k_{i}-1\right)} \end{array}$$

Where *E*_*loc,i*_ represents the local efficiency of node *i*, *k*_*i*_ represents the number of links connected to *i*, and $d_{jh}^{w}$ (*N*_*i*_) represents the weighted shortest path length between nodes *j* and *h*, which consists only of the neighbors of *i*.

The global efficiency and local efficiency were computed separately for each sparsity threshold. The area under the curve (AUC) of such metrics was then computed to produce a summarized scalar, which was independent of a specific threshold selection. The AUC values of global efficiency and local efficiency were compared between-groups using two-sample *T*-tests.

## Results

### Demographics and task behavior

The schizophrenia and healthy control groups were matched for sex (14 females for the schizophrenia group, 14 females for the healthy control group) and age (36.54 ± 8.95 years for the schizophrenia group, 36.35 ± 8.71 years for the healthy control group; *p* = 0.92). Compared with healthy controls, schizophrenia patients had significantly impaired task-switching performance, reflected in significantly increased response time [*t* (90) = 4.19, *p* < 0.001] and reduced accuracy [*t* (90) = 4.52, *p* < 0.001].

### Functional connectivity pattern and graph analysis

**FIGURE 1:**
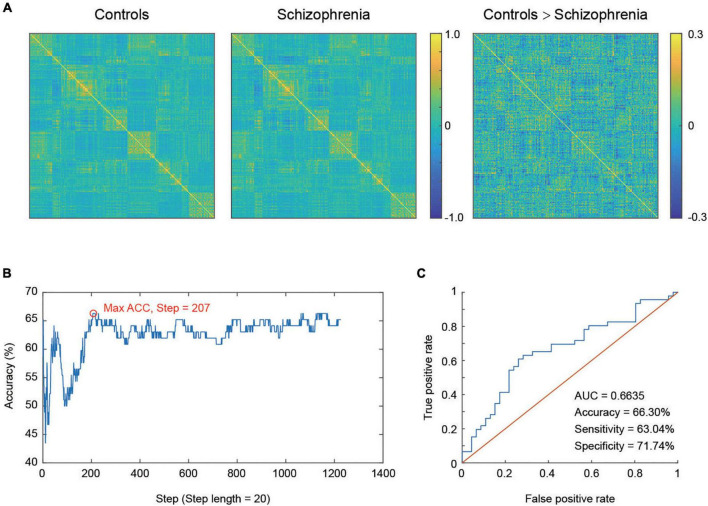
**(A)** Task-modulated connectivity matrices for the schizophrenia group and healthy control group and different connectivity matrices between these two groups. **(B)** The classification accuracy for different steps, from 20 to 24,520 with a step length of 20, the maximum ACC was obtained at step 207, which corresponds to 4,140 features. **(C)** ROC curve of the classifier.

**FIGURE 2:**
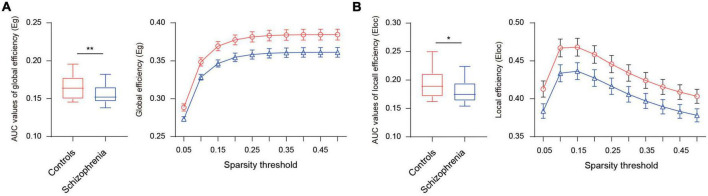
Differences in the integration and segregation of whole-brain network for the schizophrenia group and healthy control group. **(A)** The left panel displays AUC values of global efficiency, the right displays global efficiency in different sparsity thresholds (5–50% in 5% increments). **(B)** The left panel displays AUC values of local efficiency, the right displays local efficiency in different sparsity thresholds (5–50% in 5% increments). *Indicates *p* ≤ 0.05, **indicates *p* < 0.01.

To display the FC pattern, we averaged the connectivity matrix of participants within the schizophrenia and healthy control groups. Figure 1A shows the strength values of the connections between the 222 ROIs in the whole-brain in the two groups. Graph theory analysis was further used to calculate global efficiency and local efficiency to explore the distinct degree of functional integration and segregation of such a 222-node network in the two groups. We found that the global efficiency was significantly greater in the healthy controls than in the schizophrenia patients [*t* (90) = 2.69, *p* = 0.009] (Figure 2). The local efficiency was also greater in the healthy controls than in the schizophrenia patients, but this effect was marginally significant [*t* (90) = 1.95, *p* = 0.054] (Figure 2).

### Classification

Different feature numbers were first examined to determine the optimal feature size and maximize the classification accuracy. Figure 1B shows the corresponding accuracy values. The result demonstrated that the highest accuracy corresponded to 4,140 features (at step 207). In addition, the SVM classifier also collected the discriminative score of each testing participant. The receiver operating characteristic (ROC) curve of the classifier was produced using the discriminative score of each participant as a threshold. Figure 1C shows the ROC curve of the maximum accuracy condition, with AUC = 0.6635, accuracy = 66.30%, sensitivity = 63.04%, and specificity = 71.74%, which indicates that it has good classification power. The permutation test indicated that the maximum accuracy was higher than random (1,000 permutation tests, *p* < 0.001).

## Discussion

In the present study, we investigated abnormal FC patterns during task-switching in schizophrenic patients compared with healthy controls, which is related to underlying impaired cognitive flexibility in schizophrenia. Behaviorally, we found increased response time and decreased accuracy during the processing switch trial in patients with schizophrenia compared to healthy controls, revealing impairments in cognitive flexibility in schizophrenia. Multivariate pattern analysis showed that the FC pattern could distinguish schizophrenia patients and healthy controls with high classification accuracy, suggesting that the differences in the cognitive flexibility between schizophrenia patients and healthy controls involve distinct FC patterns. Moreover, the results of the graph analysis showed decreased global and local efficiency in schizophrenia patients compared to healthy controls, indicating a lower degree of network integration and segregation in schizophrenia patients during disengagement from the previous task set and engagement in an upcoming task set.

The human brain is a complex network that continuously integrates information from various brain regions associated with the neural basis of perception and cognition (11, 33, 41–43). Increasing evidence has shown that such integration (so-called FC) can be employed as a potential feature to discriminate patients from controls, which would be useful in comprehending the pathophysiology of patients with disease (12, 23, 38, 44, 45). Schizophrenia studies have employed whole-brain connectome resting-state fMRI found the altered FC which was related to cognitive deficits and negative symptoms in schizophrenia (46–48). For instance, Skudlarski et al. found that patients with schizophrenia have lower brain global connectivity which was correlated with clinical symptom severity (47). In this study, the results of brain connectome-based multivariate classifications reflected abnormal FC related to cognitive flexibility in schizophrenia patients relative to healthy controls. To the best of our knowledge, this study is the first to employ MVPA to discriminate the FC pattern related to cognitive flexibility of patients with schizophrenia from healthy controls.

Abnormal FC patterns may indicate impaired communication between distinct brain regions, potentially harming the ability to connect separate psychological and neurobiological constructs into a cohesive whole necessary for daily functioning (46). For example, schizophrenia patients present lower level of local connectedness, longer global processing length, and lower small-worldness during performing auditory oddball task when compared to the healthy control (29), suggesting that information interactions in the normal brains are more efficient at both local and global scales than in the brains with schizophrenia when performing a cognitive task. Accordingly, our results revealed that altered FC patterns during cognitive flexibility processing were driven by a lower degree of network integration and segregation in schizophrenia. This finding is consistent with previous resting-state fMRI reports (48, 49), which found significantly decreased local and global efficiency in schizophrenia. The principles of integration and segregation are fundamental to understanding dynamic network reconfigurations in the brain (31, 50, 51). Specifically, the integration of brain networks is crucial for efficient communication across entire cognitive systems (52, 53). For instance, the degree of integration typically increases when the brain processes a cognitively demanding task, which is appropriate for efficient communication among the sensory, motor and cognitive control systems (31). In contrast, the segregation of brain networks is essential for automatic (i.e., well-learned) tasks and helps to preserve resources for high-cognitive-demand events (50, 54). For instance, the degree of segregation tends to increase over time as the brain learns specialized skills, which allows the automatic processing of a habitual task without effortful cognitive control (11). To perform goal-directed behavior, the brain adjusts its network configurations (i.e., integration and segregation) to support highly efficient information transfer. Therefore, the greater integration and segmentation of brain networks is significant for improved task-switching performance. Our results indicated that both global and local information transfers at the entire-network level are less efficient in schizophrenia patients than in healthy controls during task-switching processing, giving rise to deficits in cognitive flexibility. This conclusion was also supported by other studies which have revealed that schizophrenia involves a disrupted small-world functional network characterized by reduced distributed information processing efficiency (49, 55).

The current results have a number of limitations that should be noted. First, recent network studies showed that the topological properties of the resting schizophrenic brain (e.g., global efficiency values) were positively correlated with the severity of schizophrenic symptoms (48). But we did not assess the relationship between FC alterations and clinical variables, which should be explored in future studies. Second, the sample size was relatively small to process classification. Smaller samples permit more homogeneous participants, restricting generalizability and necessitating replication (56, 57). This limitation does not influence how we interpret our findings, but it is critical to keep in mind when examining brain-behavior associations in neuroimaging research.

## Conclusion

In conclusion, we applied FC-based classification to discriminate schizophrenia patients from controls with high accuracy during task-switching processing. During task-switching processing, altered FC patterns were identified in which both global and local information transfers at the entire-network level were less efficient in schizophrenia patients than in healthy controls. These results provide insights into how the dysfunctional brain relates to abnormal cognitive flexibility induced by schizophrenia. Furthermore, we propose the use of FC as a feature to investigate the pathophysiology in schizophrenic patients.

## Data availability statement

The original contributions presented in this study are included in the article/supplementary material, further inquiries can be directed to the corresponding authors.

## Ethics statement

The studies involving human participants were reviewed and approved by the Institutional Review Boards at UCLA and Los Angeles County Department of Mental Health. The patients/participants provided their written informed consent to participate in this study.

## Author contributions

YW: data curation, formal analysis, validation, investigation, and writing—original draft. YL: writing—review and editing. XH: supervision, funding acquisition, methodology, and writing—review and editing. All authors contributed to the article and approved the submitted version.

## Footnotes

1
http://www.fil.ion.ucl.ac.uk/spm/software/spm12

2
https://www.nitrc.org/projects/gretna/

## References

[1] Uddin LQ. Cognitive and behavioural flexibility: neural mechanisms and clinical considerations. *Nat Rev Neurosci.* (2021) 22:167–79. 10.1038/s41583-021-00428-w 33536614 PMC7856857

[2] Jiang J Beck J Heller K Egner T. An insula-frontostriatal network mediates flexible cognitive control by adaptively predicting changing control demands. *Nat Commun.* (2015) 6:8165. 10.1038/ncomms9165 26391305 PMC4595591

[3] Waltz JA. The neural underpinnings of cognitive flexibility and their disruption in psychotic illness. *Neuroscience.* (2017) 345:203–17. 10.1016/j.neuroscience.2016.06.005 27282085 PMC5143214

[4] Meiran N Levine J Meiran N Henik A. Task set switching in schizophrenia. *Neuropsychology.* (2000) 14:471–82. 10.1037//0894-4105.14.3.471 10928748

[5] Jamadar S Michie P Karayanidis F. Compensatory mechanisms underlie intact task-switching performance in schizophrenia. *Neuropsychologia.* (2010) 48:1305–23. 10.1016/j.neuropsychologia.2009.12.034 20036266

[6] Greenzang C Manoach DS Goff DC Barton JJ. Task-switching in schizophrenia: active switching costs and passive carry-over effects in an antisaccade paradigm. *Exp Brain Res.* (2007) 181:493–502. 10.1007/s00221-007-0946-8 17486327

[7] Ravizza SM Moua KC Long D Carter CS. The impact of context processing deficits on task-switching performance in schizophrenia. *Schizophr Res.* (2010) 116:274–9. 10.1016/j.schres.2009.08.010 19734013 PMC2818092

[8] Livingston NR Hawkins PC Gilleen J Ye R Valdearenas L Shergill SS Preliminary evidence for the phosphodiesterase type-4 inhibitor, roflumilast, in ameliorating cognitive flexibility deficits in patients with schizophrenia. *J Psychopharmacol.* (2021) 35:1099–110. 10.1177/02698811211000778 33908296 PMC8435828

[9] Giraldo-Chica M Rogers BP Damon SM Landman BA Woodward ND. Prefrontal-thalamic anatomical connectivity and executive cognitive function in schizophrenia. *Biol Psychiatry.* (2018) 83:509–17. 10.1016/j.biopsych.2017.09.022 29113642 PMC5809301

[10] Standke I Trempler I Dannlowski U Schubotz RI Lencer R. Cerebral and behavioral signs of impaired cognitive flexibility and stability in schizophrenia spectrum disorders. *Neuroimage Clin.* (2021) 32:102855. 10.1016/j.nicl.2021.102855 34695780 PMC8551223

[11] Mohr H Wolfensteller U Betzel RF Misic B Sporns O Richiardi J Integration and segregation of large-scale brain networks during short-term task automatization. *Nat Commun.* (2016) 7:13217. 10.1038/ncomms13217 27808095 PMC5097148

[12] Rosenberg MD Finn ES Scheinost D Papademetris X Shen X Constable RT A neuromarker of sustained attention from whole-brain functional connectivity. *Nat Neurosci.* (2016) 19:165–71. 10.1038/nn.4179 26595653 PMC4696892

[13] Yang H Di X Gong Q Sweeney J Biswal B. Investigating inhibition deficit in schizophrenia using task-modulated brain networks. *Brain Struct Funct.* (2020) 225:1601–13. 10.1007/s00429-020-02078-7 32356019

[14] Li Y Wang Y Yu F Chen A. Large-scale reconfiguration of connectivity patterns among attentional networks during context-dependent adjustment of cognitive control. *Human Brain Mapp.* (2021) 42:3821–32. 10.1002/hbm.25467 33987911 PMC8288082

[15] Koch I Poljac E Muller H Kiesel A. Cognitive structure, flexibility, and plasticity in human multitasking-an integrative review of dual-task and task-switching research. *Psychol Bull.* (2018) 144:557–83. 10.1037/bul0000144 29517261

[16] Vandierendonck A Liefooghe B Verbruggen F. Task switching: interplay of reconfiguration and interference control. *Psychol Bull.* (2010) 136:601–26. 10.1037/a0019791 20565170

[17] Kiesel A Steinhauser M Wendt M Falkenstein M Jost K Philipp AM Control and interference in task switching–a review. *Psychol Bull.* (2010) 136:849–74. 10.1037/a0019842 20804238

[18] Gallos IK Mantonakis L Spilioti E Kattoulas E Savvidou E Anyfandi E The relation of integrated psychological therapy to resting state functional brain connectivity networks in patients with schizophrenia. *Psychiatry Res.* (2021) 306:114270. 10.1016/j.psychres.2021.114270 34775295

[19] Lynall ME Bassett DS Kerwin R McKenna PJ Kitzbichler M Muller U Functional connectivity and brain networks in schizophrenia. *J Neurosci.* (2010) 30:9477–87. 10.1523/JNEUROSCI.0333-10.2010 20631176 PMC2914251

[20] Whitfield-Gabrieli S Thermenos HW Milanovic S Tsuang MT Faraone SV McCarley RW Hyperactivity and hyperconnectivity of the default network in schizophrenia and in first-degree relatives of persons with schizophrenia. *Proc Natl Acad Sci U.S.A.* (2009) 106:1279–84. 10.1073/pnas.0809141106 19164577 PMC2633557

[21] Rubinov M Knock SA Stam CJ Micheloyannis S Harris AWF Williams LM Small-world properties of nonlinear brain activity in schizophrenia. *Hum Brain Mapp.* (2009) 30:403–16. 10.1002/hbm.20517 18072237 PMC6871165

[22] Sheffield JM Mohr H Ruge H Barch DM. Disrupted salience and cingulo-opercular network connectivity during impaired rapid instructed task learning in schizophrenia. *Clin Psychol Sci.* (2021) 9:210–21. 10.1177/2167702620959341 PMC10538093 37771650

[23] Yamashita M Yoshihara Y Hashimoto R Yahata N Ichikawa N Sakai Y A prediction model of working memory across health and psychiatric disease using whole-brain functional connectivity. *Elife.* (2018) 7:e38844. 10.7554/eLife.38844 30526859 PMC6324880

[24] Rissman J Gazzaley A D’Esposito M. Measuring functional connectivity during distinct stages of a cognitive task. *Neuroimage.* (2004) 23:752–63. 10.1016/j.neuroimage.2004.06.035 15488425

[25] Bassett DS Bullmore E Verchinski BA Mattay VS Weinberger DR Meyer-Lindenberg A. Hierarchical organization of human cortical networks in health and schizophrenia. *J Neurosci.* (2008) 28:9239–48. 10.1523/JNEUROSCI.1929-08.2008 18784304 PMC2878961

[26] Li M Chen Z Li T. Small-world brain networks in schizophrenia. *Shanghai Arch Psychiatry.* (2012) 24:322–7. 10.3969/j.issn.1002-0829.2012.06.003 25324636 PMC4198898

[27] van Diessen E Diederen SJ Braun KP Jansen FE Stam CJ. Functional and structural brain networks in epilepsy: what have we learned? *Epilepsia.* (2013) 54:1855–65. 10.1111/epi.12350 24032627

[28] Dehaene S Naccache L. Towards a cognitive neuroscience of consciousness: basic evidence and a workspace framework. *Cognition.* (2001) 79:1–37. 10.1016/s0010-0277(00)00123-2 11164022

[29] Ma S Calhoun VD Eichele T Du W AdalD TC. Modulations of functional connectivity in the healthy and schizophrenia groups during task and rest. *Neuroimage.* (2012) 62:1694–704. 10.1016/j.neuroimage.2012.05.048 22634855 PMC3408853

[30] Yin S Li Y Chen A. Functional coupling between frontoparietal control subnetworks bridges the default and dorsal attention networks. *Brain Struct Funct.* (2022) 227:2243–60. 10.1007/s00429-022-02517-7 35751677

[31] Keerativittayayut R Aoki R Sarabi MT Jimura K Nakahara K. Large-scale network integration in the human brain tracks temporal fluctuations in memory encoding performance. *Elife.* (2018) 7:e32696. 10.7554/eLife.32696 29911970 PMC6039182

[32] Poldrack RA Congdon E Triplett W Gorgolewski KJ Karlsgodt KH Mumford JA A phenome-wide examination of neural and cognitive function. *Sci Data.* (2016) 3:160110. 10.1038/sdata.2016.110 27922632 PMC5139672

[33] Power JD Cohen AL Nelson SM Wig GS Barnes KA Church JA Functional network organization of the human brain. *Neuron.* (2011) 72:665–78. 10.1016/j.neuron.2011.09.006 22099467 PMC3222858

[34] Cole MW Reynolds JR Power JD Repovs G Anticevic A Braver TS. Multi-task connectivity reveals flexible hubs for adaptive task control. *Nat Neurosci.* (2013) 16:1348–55. 10.1038/nn.3470 23892552 PMC3758404

[35] Mumford JA Turner BO Ashby FG Poldrack RA. Deconvolving BOLD activation in event-related designs for multivoxel pattern classification analyses. *Neuroimage.* (2012) 59:2636–43. 10.1016/j.neuroimage.2011.08.076 21924359 PMC3251697

[36] Chang C-C Lin C-J. LIBSVM: a library for support vector machines. *ACM Trans Intell Syst Technol.* (2011) 2:1–27. 10.1145/1961189.1961199

[37] Chen Y-W Lin C-J. Combining SVMs with various feature selection strategies. In: Guyon I Nikravesh M Gunn S Zadeh LA editors. *Feature Extraction: Foundations and Applications.* Berlin: Springer (2006). p. 315–24.

[38] Liu F Guo W Fouche J-P Wang Y Wang W Ding J Multivariate classification of social anxiety disorder using whole brain functional connectivity. *Brain Struct Funct.* (2015) 220:101–15. 10.1007/s00429-013-0641-4 24072164

[39] Akay MF. Support vector machines combined with feature selection for breast cancer diagnosis. *Expert Syst Appl.* (2009) 36:3240–7. 10.1016/j.eswa.2008.01.009

[40] Golland P Fischl B. Permutation tests for classification: towards statistical significance in image-based studies. *Inf Process Med Imaging.* (2003) 18:330–41. 10.1007/978-3-540-45087-0_28 15344469

[41] Cole MW Bassett DS Power JD Braver TS Petersen SE. Intrinsic and task-evoked network architectures of the human brain. *Neuron.* (2014) 83:238–51. 10.1016/j.neuron.2014.05.014 24991964 PMC4082806

[42] Cocchi L Zalesky A Fornito A Mattingley JB. Dynamic cooperation and competition between brain systems during cognitive control. *Trends Cogn Sci.* (2013) 17:493–501. 10.1016/j.tics.2013.08.006 24021711

[43] Bassett DS Sporns O. Network neuroscience. *Nat Neurosci.* (2017) 20:353–64. 10.1038/nn.4502 28230844 PMC5485642

[44] Yang M Li J Li Z Yao D Liao W Chen H. Whole-brain functional connectome-based multivariate classification of post-stroke aphasia. *Neurocomputing.* (2017) 269:199–205. 10.1016/j.neucom.2016.10.094

[45] Zhong X Shi H Ming Q Dong D Zhang X Zeng L-L Whole-brain resting-state functional connectivity identified major depressive disorder: a multivariate pattern analysis in two independent samples. *J Affect Disord.* (2017) 218:346–52. 10.1016/j.jad.2017.04.040 28499208

[46] Hummer TA Yung MG Goñi J Conroy SK Francis MM Mehdiyoun NF Functional network connectivity in early-stage schizophrenia. *Schizophr Res.* (2020) 218:107–15. 10.1016/j.schres.2020.01.023 32037204

[47] Skudlarski P Jagannathan K Anderson K Stevens MC Calhoun VD Skudlarska BA Brain connectivity is not only lower but different in schizophrenia: a combined anatomical and functional approach. *Biol Psychiatry.* (2010) 68:61–9. 10.1016/j.biopsych.2010.03.035 20497901 PMC2900394

[48] Su TW Hsu TW Lin YC Lin CP. Schizophrenia symptoms and brain network efficiency: a resting-state fMRI study. *Psychiatry Res.* (2015) 234:208–18. 10.1016/j.pscychresns.2015.09.013 26409574

[49] Liu Y Liang M Zhou Y He Y Hao Y Song M Disrupted small-world networks in schizophrenia. *Brain.* (2008) 131(Pt 4):945–61. 10.1093/brain/awn018 18299296

[50] Cohen JR D’Esposito M. The segregation and integration of distinct brain networks and their relationship to cognition. *J Neurosci.* (2016) 36:12083–94. 10.1523/JNEUROSCI.2965-15.2016 27903719 PMC5148214

[51] Shine JM Poldrack RA. Principles of dynamic network reconfiguration across diverse brain states. *Neuroimage.* (2018) 180(Pt B):396–405. 10.1016/j.neuroimage.2017.08.010 28782684

[52] Sadaghiani S Poline JB Kleinschmidt A D’Esposito M. Ongoing dynamics in large-scale functional connectivity predict perception. *Proc Natl Acad Sci U.S.A.* (2015) 112:8463–8. 10.1073/pnas.1420687112 26106164 PMC4500238

[53] Bassett DS Wymbs NF Rombach MP Porter MA Mucha PJ Grafton ST. Task-based core-periphery organization of human brain dynamics. *PLoS Comput Biol.* (2013) 9:e1003171. 10.1371/journal.pcbi.1003171 24086116 PMC3784512

[54] Bassett DS Yang M Wymbs NF Grafton ST. Learning-induced autonomy of sensorimotor systems. *Nat Neurosci.* (2015) 18:744–51. 10.1038/nn.3993 25849989 PMC6368853

[55] Bassett DS Bullmore ET Meyer-Lindenberg A Apud JA Weinberger DR Coppola R. Cognitive fitness of cost-efficient brain functional networks. *Proc Natl Acad Sci U.S.A.* (2009) 106:11747–52. 10.1073/pnas.0903641106 19564605 PMC2703669

[56] Schnack HG Kahn RS. Detecting neuroimaging biomarkers for psychiatric disorders: sample size matters. *Front Psychiatry.* (2016) 7:50. 10.3389/fpsyt.2016.00050 27064972 PMC4814515

[57] Beleites C Neugebauer U Bocklitz T Krafft C Popp J. Sample size planning for classification models. *Anal Chim Acta.* (2013) 760:25–33. 10.1016/j.aca.2012.11.007 23265730

